# Timing and Outcomes of Cranioplasty After Decompressive Craniectomy: A Systematic Review of Neurological Recovery, Complications, and Predictive Factors

**DOI:** 10.3390/jcm15082813

**Published:** 2026-04-08

**Authors:** András Gati, Árpád Viola, Yousif Qais Al-Khafaji, Siran Aslan, Mustafa Qais Al-Khafaji, Yousif Asaad Taha, Murtadha Qais Al-Khafaji, Georgia Koudigkeli, Shahad Qais Al-Khafaji, Mohammad Walid Al-Smadi

**Affiliations:** 1 Department of Neurosurgery, Dr. Manninger Jenő Traumatology Institute, 1081 Budapest, Hungaryarpadviola@gmail.com (Á.V.); shahadqais@gmail.com (S.Q.A.-K.); 2 Neurotraumatology Division, Semmelweis University, 1081 Budapest, Hungary; drsiran.5@gmail.com; 3 Faculty of Medicine, University of Debrecen, 4032 Debrecen, Hungary; yousifqais7@hotmail.com; 4 Doctoral School of Clinical Medicine, Semmelweis University, 1083 Budapest, Hungary; 5 Department of Plastic Surgery, Norfolk and Norwich University Hospital, Norwich NR4 7UY, UK; mustafa.al-khafaji@nhs.net; 6 Department of Neurology, Siofok Korhaz Rendelointezet, 1125 Budapest, Hungary; yousif1999asaad@gmail.com; 7 Department of Trauma and Orthopaedics, Frimley Park Hospital, Frimley GU16 7UJ, UK; murtadha.al-khafaji1@nhs.net; 8 Brighton and Sussex Medical School, Brighton BN1 9PX, UK; g.koudigkeli1@uni.bsms.ac.uk

**Keywords:** cranioplasty, decompressive craniectomy, timing, neurological outcome, complications, traumatic brain injury

## Abstract

**Background:** The optimal timing of cranioplasty (CP) after decompressive craniectomy (DC) remains debated. Early reconstruction may enhance neurological recovery through restoration of cerebral perfusion and cerebrospinal fluid dynamics, yet concerns persist regarding postoperative complications. Objective: To evaluate the impact of early versus delayed cranioplasty on neurological outcomes and postoperative complications in adults following decompressive craniectomy. **Methods:** A systematic review was conducted in accordance with PRISMA guidelines (PROSPERO ID: CRD420251123808). PubMed, OVID, and Web of Science were searched for studies published between January 2017 and December 2025. Eligible studies compared early and delayed CP in adults and reported neurological outcomes and/or complications. **Results:** Twenty-one retrospective cohort studies including 8462 patients were analyzed. Neurological improvement was observed in both groups across multiple validated scales (GOSE, GOS, GCS, mRS, BI, FIM, NIHSS, MMSE). Early CP was consistently associated with superior recovery, including higher one-year Barthel Index improvement (74.1% vs. 54.8%), greater FIM gains (7.31% vs. 4.66%), and higher composite recovery rates (95.6% vs. 80.0%). No study demonstrated superior recovery with delayed CP. Infection, hydrocephalus, and seizure rates were comparable between groups. However, hematoma (21% vs. 10.4%) and hygroma (7.49% vs. 4.73%) were more frequent after early CP, although hematoma rates were influenced by a large database study. Bone flap resorption was less frequent with early CP (1.44% vs. 6.26%). **Conclusions:** Early cranioplasty is associated with improved neurological recovery but carries an increased risk of select complications, particularly hematoma and hygroma, representing a clinically relevant trade-off. Delayed CP does not demonstrate overall superior safety due to higher bone flap resorption. Timing should be individualized, and prospective multicenter studies with standardized definitions are needed.

## 1. Introduction

Cranioplasty (CP) is a routinely performed neurosurgical procedure aimed at restoring cranial integrity following decompressive craniectomy (DC) [1]. DC is commonly indicated for the management of refractory intracranial hypertension due to traumatic brain injury (TBI) or cerebrovascular conditions [2]. Reconstruction of the cranial defect is typically achieved using either the preserved autologous bone flap or patient-specific synthetic implants [3].

Beyond its reconstructive role, CP has important physiological and neurological implications. It has been associated with restoration of cerebrospinal fluid (CSF) dynamics, improvement in cerebral perfusion, normalization of intracranial pressure relationships, and enhancement of neurological recovery [1,4]. However, this procedure is associated with significant morbidity, including hematoma, infections, hydrocephalus, seizures, and even death [5]. The complication rate is reported to be up to 35%, with infection being the most common in the postoperative period [3]. These complications are often associated with patient demographics, existing medical conditions, indications of CP, the surgical procedure itself, and the underlying disease [4,5]. With improved survival rates following DC, the number of subsequent CP procedures is increasing, highlighting the importance of optimizing this procedure [2].

The optimal timing of CP after DC has been a subject of debate. Traditionally, surgeons have delayed the procedure for several months to allow recovery and resolution of cerebral edema. However, recent evidence suggests that early CP (within 3 months of DC) has gained support as a safe option that could potentially improve neurological recovery [1,6,7,8].

Despite this growing interest, the literature remains heterogeneous and at times conflicting, particularly regarding complication rates and functional outcomes. Variability in study design, patient populations, definitions of timing, and outcome measures further complicates interpretation and limits the ability to draw definitive conclusions [2].

Therefore, this systematic review aims to evaluate the impact of CP timing on neurological outcomes and postoperative complications, comparing early (≤3 months) versus delayed (>3 months) cranioplasty. In addition, we examine the methods used to assess neurological recovery, the influence of surgical and material-related factors, and the role of advanced imaging modalities, including functional and diffusion MRI, in evaluating postoperative outcomes.

## 2. Materials and Methods

This systematic review was conducted following the Preferred Reporting Items for Systematic Reviews and Meta-Analyses (PRISMA) guidelines to ensure comprehensive reporting and methodological rigor [9]. The PRISMA checklist is provided in File S1.

### 2.1. Research Aim and Search Strategy

**Figure 1:**
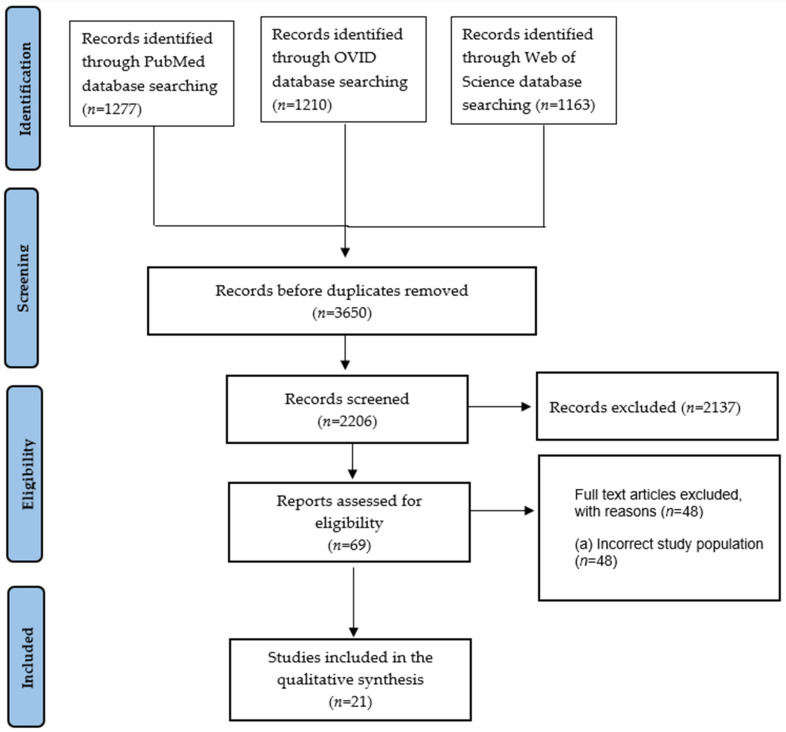
Schematic representation of study selection based on PRISMA.

The studies included in this systematic review were selected using an adapted PRISMA framework, as depicted in Figure 1. The study was registered on PROSPERO ID: [CRD420251123808]. A preliminary protocol guided the literature search, with studies being screened based on the following criteria:

- P ex (Population): Patients aged 18 and above with large cranial defects following decompressive craniectomy.
- I (Intervention): Patients who underwent early and late cranioplasty after decompressive craniectomy.
- C (Comparison): Compare postoperative complications and other findings after CP between patients in the early and late groups.
- O (Outcomes): Success rate, complication rates, patient survival, neurological function recovery, recovery time.

The databases used in this research were PubMed, OVID, and Web of Science, covering studies from 2017 to 2025, using search criteria incorporating MESH terms (Table 1).

### 2.2. Selection Criteria

Inclusion and exclusion criteria were developed collaboratively by the research team to ensure comprehensive, methodologically sound study selection and data extraction. Inclusion criteria encompassed original research, cohort studies, retrospective studies, prospective studies, randomized controlled trials (RCTs), and case series on early and late CP Following DC in the past 8 years (2017–2025). Exclusion criteria involved narrative reviews, editorials, letters, comments, protocols, guidelines, case reports, non-English language scientific articles, and articles for which the full text was unavailable. Furthermore, articles on pediatric patients and articles not relevant to the research question were excluded.

### 2.3. Data Extraction and Management

A standard template, based on the Cochrane Consumers and Communication Review group’s extraction template, facilitated data extraction for quality assessment and evidence synthesis. Extracted data included author names, database, journal, publication date, article type, DOI, titles, abstracts, applied methodology, and results. Two review authors independently screened each record by title and abstract, with a third reviewer resolving any conflicts. Any discrepancies were discussed and resolved with the two remaining authors as necessary. Full texts were reviewed for all potentially relevant studies, and final decisions on inclusion were made independently. A narrative summary was prepared for the studies included in the review based on the success rate, complication rates, patient survival, neurological function recovery, and recovery time. Any study providing information outside these groups was categorized as miscellaneous and summarized in the subsequent text.

### 2.4. Risk of Bias Assessment

The Newcastle–Ottawa Scale (NOS) for observational studies was used to independently assess the methodological quality of the included studies. The NOS evaluates studies across three domains: group comparability, outcome or exposure analysis, and study group selection. The selection domain was given a maximum of 4 stars, the comparison domain 2 stars, and the outcome domain 3 stars. Studies with 7–9 stars were classified as “low risk of bias,” studies with 5–6 stars as “moderate risk of bias,” and studies with fewer than five stars as “high risk of bias,” ensuring a complete examination. 13 included studies had moderate risk of bias, while 8 studies had low risk of bias.

### 2.5. Structure Overview

Due to substantial heterogeneity in study design, outcome measures, and timing, a meta-analysis was not performed. A structured narrative synthesis was conducted, grouping studies by neurological outcomes and complication profiles to enable comparison between early and delayed cranioplasty. Studies were grouped for synthesis by cranioplasty timing (early ≤3 months vs. delayed >3 months), and each group was further divided into subsections discussing outcomes assessed using different evaluation tools. Outcomes were summarized using descriptive statistics, including proportions and mean differences, where reported. This structure allows a focused comparison of the efficacy and safety profiles of each timing approach on post-CP outcomes.

## 3. Results

### 3.1. Demographic and Clinical Data of Radiology

Initially, our search strategy yielded 3650 studies collected from the three databases mentioned above (PubMed: 1277, OVID: 1210, Web of Science: 1163). A total of 21 articles met the eligibility criteria and were reviewed after thorough abstract screening and duplicate removal (Figure 1). They comprise 20 cohort retrospective studies and 1 retrospective propensity-matched national database cohort study [10]. Three studies were multicenter [11,12,13,14] and the remaining studies were conducted at a single institution.

A total of 8462 patients were included in those studies. The early patients had a mean age of around 46 years, and the late group had a collective mean age of around 46 years. Overall, the mean age of all patients was 45 years. Three studies did not mention the mean age of the patients [10,15,16]. Only three papers did not describe the genders [10,17,18] Three studies did not specifically mention the period of follow-up for the cases [15,16,19]. The most common cause of decompressive craniectomy in our review was trauma.

Among the included studies, 15 articles used computed tomography (CT) scans to assess structural and pathological changes associated with CP Postoperatively [11,12,13,14,15,16,18,19,20,21,22,23,24,25,26]. In one study, metabolic brain activity related to neurological recovery was evaluated using Positron Emission Tomography (PET) [17]. Additionally, no radiological imaging techniques were used in four studies to assess the results of cranioplasty [10,27,28,29].

In these papers, CT imaging was commonly used to assess the presence of intracranial hypertension, reflected by midline shift, and to identify various types of intracranial hematomas as well as features of the syndrome of the trephined (sunken brain syndrome). One study specifically used CT to evaluate for compression or absence of basal cisterns, which serve as indicators of increased intracranial pressure or mass effect [13].

One study utilized CT scans at the time of admission to assess for ventriculomegaly as a sign of hydrocephalus [26]. In another study, CT was employed to detect edema surrounding the CP site as a possible marker of infection and to evaluate bone flap resorption (BFP) [15]. Overall, these radiological parameters were essential in identifying complications, monitoring postoperative progress, and assessing outcomes following early versus late CP.

### 3.2. Indications of Cranioplasty Timing

The timing of cranioplasty (CP) following decompressive craniectomy (DC) is variable; decisions are typically based on surgeon preference and patient-specific factors such as age, etiology, and recovery. Some studies agree on general prerequisites for any timing indication, such as the resolution of cerebral edema, a well-healed surgical site, comorbidities and systemic diseases, restoration of normal intracranial pressure, and overall clinical stability [10,11,14,15,16,20,21,22,27]. Other early repair indications include improving cerebral blood flow and CSF hydrodynamics, and avoiding “syndrome of the trephined” and hydrocephalus [10,12,14,16,18,19,20,23,25,26,29]. From a surgical perspective, early timing allows for easier tissue dissection before severe adhesions form, contributing to a shorter surgery duration [11,16,20,26]. Early-phase CP was indicated to reduce psychological stress associated with prolonged skull defects, while protecting brain tissue stability from external atmospheric and gravitational forces [11,29].

Conversely, indications for delaying CP are historically rooted in safety and in reducing infection risk [10,20,24,25]. Additionally, three studies note that in severe trauma cases, delay is indicated to maintain access for potential salvage surgery and to allow time for patient recovery [14,25,29]. Aesthetically, late CP was indicated to prevent poor scalp growth [16]. One study identifies logistics as a factor leading to late cranioplasty, noting that some trauma patients simply do not return expediently for the procedure [23].

Beyond clinical factors, non-clinical logistics also play a significant role in determining CP timing, including hospital costs, the availability of customized plates, inadequate ambulatory care, and social factors such as operative convenience or family preference [12,21,22,23,25,26]. One study noted a selection bias, as the majority of patients receiving early cranioplasty had trauma as their underlying pathology. In contrast, stroke patients were often transferred to local stroke units later [22].

### 3.3. Demographic and Clinical Data of Patients Using Neurological Assessment Tools

A review of 20 studies found that 10 articles used at least one functional neurological assessment tool [11,12,13,16,17,18,20,24,27,29], while 10 focused solely on the clinical complications. These studies were all retrospective, and a total of 8462 patients were included in the analysis.

#### 3.3.1. GOSE as a Neurological Assessment Tool

The GOSE was used in two studies to summarize neurological findings in individuals after CP [12,13]. Both articles included 299 patients, divided into an early CP group of 140 and a late CP group of 159. Post-op GOSE scores ranged from 2 to 6 in both groups, with a median of 4 at follow-up. Both groups were followed for 12 months and showed neurological improvement. A summary of these findings is provided in Table 2.

#### 3.3.2. GCS as a Neurological Assessment Tool

One retrospective study used the GCS to assess 138 patients across five groups based on the time interval between DC and CP [11]. Among the patients, 94 males and 44 females aged 34–67 years were involved and were followed up for an average of 1 month. Although there was no significant difference between preoperative and postoperative GCS scores, both the early and late CP groups showed clinical improvement. Additionally, this article reported that early CP was associated with a lower risk of midline shift and complication rates, due to its positive effects on CSF circulation and cerebral blood flow [2]. (Table 3)

#### 3.3.3. GOS as a Neurological Assessment Tool

In one retrospective study, functional outcome at 12 months following decompressive craniectomy was evaluated using the Glasgow Outcome Scale (GOS) based on the time of the CP [18]. In total, 55 patients were enrolled in the study; they were split into two groups: early CP (<3 months; *n* = 31) and late CP (≥3 months; *n* = 24). The early CP group (14/31, 45.16%) had a smaller proportion of patients with an adverse 12-month outcome (GOS 1–3) than the late CP group (19/24, 79.17%). However, late cranioplasty was not an independent predictor of a less favorable functional outcome in multivariate analysis (OR 1.21, 95% CI 0.45–3.28; *p* = 0.703). Similarly, 5-year mortality was numerically lower in the early CP group (20 deaths) than in the late CP group (17 deaths).

#### 3.3.4. FIM as a Neurological Assessment Tool

Only one study reported using the Functional Independence Measure (FIM) scale in a cohort of 31 patients. The study population consisted of 16 males and 15 females. Of the 16 patients who underwent early CP, 12 demonstrated postoperative improvement, with the mean preoperative FIM score increasing from 70.06 to 77.37. On the other hand, for the late CP group, 8 out of 15 patients showed improvement, with the mean preoperative score increasing from 68.40 to 73.07. FIM scores were assessed seven days before and thirty days after surgery. Across both groups, patients either demonstrated improved FIM scores or maintained their preoperative values. Comorbidities were not reported in this study (Table 4).

#### 3.3.5. BI as a Neurological Assessment Tool

One article reported the results of 100 patients assessed by the Barthel Index (BI). Before CP, there was no significant difference in the BI scores between the early and late CP groups. However, after 1 year, patients in the early CP group showed a significant improvement in BI scores, whereas those in the late CP group did not. Additionally, a significantly higher proportion of patients in the early CP group showed neurological functional improvement (74.1% vs. 54.8%). In contrast, a larger proportion of patients in the late CP group maintained neurological stabilization (42.9% vs. 24.1%), with only a small number of patients in both groups showing deterioration (Table 5).

#### 3.3.6. Combined Neurological Assessment Tools

##### NIHSS, MMSE, NCSE, FIM, GCS, Stress as Neurological Assessment Tools (Six Tools)

One study used six approaches for investigating neurological outcomes following CP (GCS, NIHSS, FIM, MMSE, NCSE, and stress markers). A total of 90 patients were included, with 45 in the early group (<3 months) and 45 in the delayed group (3–6 months). Clinical efficacy was determined by GCS, with a recovery rate of 95.6% in the early group compared to 80% in the delayed group. The early group had significantly better neurological function as measured by the NIHSS scores. Cognitive function, as evaluated with FIM, MMSE, and NCSE, improved in both groups, with consistently higher scores in the early CP group. Stress markers (IL-6, cortisol, TNF-α) increased after treatment in both groups, although the early group showed much lower levels. Patient details and clinical data are presented in Table 6.

##### KPS, ZPS, Quality of Life, Psychological Function as Neurological Assessment Tools (Four Tools)

One study used four tools (KPS, ZPS, psychological function, and quality of life) to assess the outcome of CP in 60 patients with TBI, who were categorized into two groups. The super-early repair group consisted of 28 patients (17 men, 11 women) aged 17–65 years who underwent surgery within 4–6 weeks of DC. The conventional repair group comprised 32 cases (26 males and 6 females) who underwent surgery 3–6 months after DC. However, this study failed to evaluate the comorbidities and the follow-up period.

The postoperative outcomes favored the super-early group across most measures. In total, 17 patients in the super-early group achieved a KPS score above 50, compared with 15 in the conventional group, and good ZPS scores were observed in 17 patients in the early group and 15 in the conventional group. Good psychological function was reported by 23 patients in the 4–6 weeks group and 13 cases in the 3–6 months group. Moreover, a good quality of life was observed in 23 patients in the earlier CP group compared with 18 in the later CP group. Overall, earlier CP using these four neurological assessment measures was associated with improved functional, psychosocial, and quality-of-life outcomes (Table 7).

##### MMSE, PGIBBD, GOS as Neurological Assessment Tools (Three Tools)

Only one study used three methods to assess cognitive and functional outcomes in patients following early or late CP (MMSE, PGIBBD, and GOS). For MMSE and PGIBBD, 38 patients were selected (19 per group), and both groups showed postoperative improvement, with slightly greater improvement in the late CP cohort. A total of 60 patients were examined using GOS (30 in each group), and functional recovery improved in both groups during a 3-month follow-up period. Patient details and clinical data are presented in Table 8.

##### GOS, mRS as Neurological Assessment Tools (Two Tools)

Finally, one study used two tools (GOS and mRS) to evaluate outcomes following CP. A total of 101 patients were included, with 41 in the early group (<3 months) and 60 in the late group (>3 months). The early group had a mean age of 32 years, while the late group had a mean age of 31 years. A total of 17 patients across both groups had comorbidities, but the specific types were not mentioned. Both groups showed similar postoperative improvements in GOS and reduction in mRS. However, the late group had a longer follow-up period (582.6 vs. 315.6 days). Patient details and clinical data are presented in Table 9.

### 3.4. Complications as an Assessment Tool

Finally, we analyzed data from 21 studies involving 7921 of 8462 patients; the remaining patients were excluded due to unavailable complication data. The patients were divided into two groups: the early group included 3568 patients, and the late CP group included 4353. Overall complications and patient demographic clinical data are provided in Table 10. The complication rate did not differ much between the early and late groups. The frequency of complications in the early group was 2040 in 3568 patients (57.2%), whereas the late group had 1673 complications in 4353 patients (38.4%). Most complications were reported in the national database study [10].

#### 3.4.1. Infection

Fifteen studies [10,14,15,16,20,21,22,23,25,29,30] reported infection as a complication; however, four of these studies could not differentiate the complication between the early and late groups [11,19,26,28]. A total of 6285 patient outcomes were included. The incidence of infections in the early group was 9.11% (*n* = 271/2974) and 5.56% (*n* = 184/3311) in the late group. Abscesses and granulomas were reported in 89 patients in the early group, while in the late group, they were observed in 80 patients [10,22]. No statistically significant difference was found between the two groups.

#### 3.4.2. Hydrocephalus

Ten studies reported hydrocephalus as a complication [10,12,13,14,20,22,23,24,27,30]. Data from 6216 patients were analyzed, and the occurrence of hydrocephalus was 7.53% (*n* = 228/3026) in the early group, and 7.13% (*n* = 226/3200) in the late group.

#### 3.4.3. Hematoma

Twelve studies [10,14,16,19,20,21,22,24,26,28,29,30] provided data about postoperative hematoma (ICH, EDH, SDH). The incidence of hematoma was 21.0% (*n* = 612/2911) in the early group and 10.4% (*n* = 311/3003) in the late group. Three articles reported hematoma as one of the complications post-CP, but they did not state whether they occurred in the early or late group [19,26,28].

#### 3.4.4. Seizure

Six studies provided data about seizure incidence in 5827 patients [10,12,24,27,30]. The combined seizure rate was 11.5% (*n* = 669/5827). The early group reported seizures as the outcome in 10.5% (*n* = 302/2868), and the late group reported 12.4% (*n* = 367/2959).

#### 3.4.5. Bone Graft Resorption (BGR)

Five studies reported BGR or sunken brain syndrome (syndrome of the trephined) outcomes in 1724 patients [14,20,21,23,27], resulting in a combined incidence of 6.26% (*n* = 108/1724). The early group reported 1.44% (*n* = 12/832), while the late group reported 11% (*n* = 98/892).

#### 3.4.6. Hygroma

Only two studies reported hygroma in 1461 patients [14,27]. Fifty-four out of 721 patients (7.49%) in the early CP group complained of hygroma as a complication, while the late group reported only 35 hygroma cases out of 740 patients (4.73%).

#### 3.4.7. Other Complications

Only 24 mortalities were reported in the early CP group [10,27], while one study reported 20/31 cases of mortality after 5 years in the early CP group, and 17/24 in the delayed CP group [18]. One patient in the early group had a reported case of pneumocephalus, while one patient in the late group had a cosmetic defect [22]. Only one case of wound dehiscence was identified in the early group, compared with nine patients in the late group [23]. Extra-axial collections (EACs) occurred in six patients in the early group, compared with only one in the late CP group [23]. In the early CP group, 55 patients experienced impaired wound healing [10,24], whereas 49 patients in the late CP group experienced wound disruption [10]. Fluid buildup or effusion was found in 36 people in the early group and 17 patients in the late group [10,16,29,30]. For wound necrosis, six cases were reported in the early group and 11 in the late group [16,29,30]. Only one patient in the early group complained of a chewing problem, while two people receiving late CP experienced chewing issues [29]. A dural tear was reported in only one study; Yan et al., [30] reported that two patients in the early group and six patients in the late group experienced a dural tear. CP Reoperation was necessary in 10 patients (19.6%) in the early group and 6 patients (6.60%) in the late CP group [23]. Craniectomy/craniotomy reoperation was done for 74/680 patients in the early group, and for 55/680 patients in the late CP group [10]. One study reported that 79 patients in the early group had to depend on a wheelchair/care provider, while the late group included 69 patients [10]. Removal/replacement of the bone flap occurred in 66 patients in the early group and in 68 patients in the late CP group [10,22].

## 4. Discussion

This systematic review assessed whether the timing of cranioplasty after DC influences neurological recovery and complications. Across the 21 included cohort studies, neurological function improved after CP, irrespective of timing, with multiple reports suggesting that earlier intervention (≤3 months) can accelerate or amplify recovery without a clear penalty in adverse events [11,12,13,16,17,22,23,28,29]. These findings are consistent with prior literature indicating that CP reverses “syndrome of the trephined,” normalizes CSF hydrodynamics, and improves cerebral perfusion/metabolism [1,2,6,7,8,12,17,23,29].

### 4.1. Neurological Recovery

Functional scales used across studies (GOSE, GOS, mRS, BI, FIM, NIHSS, MMSE, NCSE, kPS/ZPS) showed postoperative improvement in both early and late groups, with several advantages for earlier repair. Zhao et al. demonstrated higher 12-month BI in earlier CP [22], Tomar et al. reported larger 30-day FIM gains with early timing [11], and Li et al. found better composite neurological and cognitive metrics (GCS, NIHSS, MMSE, NCSE, FIM) and attenuated stress responses in early groups [29].

Our results are consistent with previous meta-analyses that found that early CP was associated with better functional recovery [1,2,6,7]. Malcolm et al. [1] found that both early and late CP led to significant neurological improvement. However, the neurological outcome was significantly better in the early cohort than in the late cohort. In addition, De Cola et al. analyzed pre- and post-CP outcomes. They found that performing the procedure within 90 days of DC was associated with greater improvement in motor performance, while no significant differences were observed in MMSE or memory function scores [31]. Although a higher incidence of hydrocephalus was noted in early patients, Vreeburg et al. more recently found no significant difference in functional outcome (GOSE) between early (≤90 days) and late (>90 days) CP [12]. Finally, while acknowledging that benefits may vary by timing thresholds and patient subgroups, these more recent findings support the clinical benefits of early CP [32].

### 4.2. Complication Rates

Complications, however, are a significant issue, with rates ranging from 38 to 57% [10,11,12,13,14,15,16,17,18,19,20,21,22,23,24,25,26,27,28,29,30]. In our study, the frequency of complications in the early group was 57.2%, while the late group had 38.4%. For this review, infection, hydrocephalus, and seizures were noted at similar frequencies in the early and late groups, as noted by previous systematic reviews [2,4].

Infection, one of the most frequent complications, can be related to timing (9.11% in the early group and 5.56% in the late group) [10,14,15,16,20,21,22,23,25,29,30]. Chasles et al. reported similar infection rates after the procedure and stated that storage of bone grafts and the use of preventive antibiotics may alter the incidence [33]. Another interesting finding was that subcutaneous preservation or cryopreservation of the bone flap did not significantly alter infection rates [34].

Hydrocephalus was noted in ~7% of cases, and there was some evidence that early CP would decrease its occurrence by normalizing CSF flow [23], although other groups noted the opposite [13]. A 2023 meta-analysis indicated that CP within <35 days can potentially decrease subdural effusion but elevated the risk of hydrocephalus [35]. Malcolm et al. (6%) and Kurland et al. (7.5%) reported a lower rate of hydrocephalus, and their studies indicated that early CP increases the odds of hydrocephalus [1,36]. They also state that it is hard to identify whether it is due to the initial brain insult, craniectomy, or CP itself [1,36,37].

An additional important consideration is the presence of an extracranial herniation associated with hydrocephalus. In such cases, elevated intracranial pressure and outward brain expansion through the craniectomy defect may complicate early reconstruction. This phenomenon may necessitate delaying cranioplasty until CSF diversion or adequate intracranial pressure control is achieved. Therefore, hydrocephalus accompanied by extracranial herniation represents not only a complication but also a potential contraindication to early cranioplasty in selected patients.

In this review, we reported a hematoma rate of 21.0% in the early group and 10.4% in the late group. Seizures occurred 10.5% of the time during early CP, and the late group reported 12.4%. This differs from other studies, where seizures present at much lower rates between 5.9% and 6.6%, and early groups noted at higher incidences. They also conclude that seizure incidence data must be interpreted with caution, as retrospective analyses typically underestimate seizure occurrence compared to prospective investigations. Notably, the frequency of new-onset seizures following CP does not appear to differ across DC indications [33,37].

The occurrence of BGR and sunken brain syndrome in our study was 6.26% in the early group and 1.44% in the late group. Kurland et al. reported that aseptic bone flap resorption may occur in up to 16% of adult patients, while cosmetic irregularities such as flap depression were observed in approximately 3.1% of cases [36]. Malcolm et al. reported an incidence of aseptic bone flap resorption of 10.8% in adult patients. They observed that resorption occurred substantially more often in the pediatric population, particularly when CP was delayed beyond six weeks. Among adults, however, their analysis showed no significant difference in the odds of bone flap resorption between procedures performed before and after 12 weeks [37].

We found that 7.49% of patients in the early CP group complained of hygroma, while the late group only reported 4.73%. This higher incidence in the early cohort may be explained by incomplete normalization of cerebrospinal fluid dynamics and brain compliance at the time of reconstruction. Residual cerebral edema and impaired CSF absorption may contribute to subdural fluid accumulation, while early restoration of cranial vault integrity can alter intracranial pressure gradients and promote hygroma formation. Fluid buildup or effusion was found in 4.56% in the early group and 2.11% in the late group. Chasles et al. found higher pooled rates of 4.2%, with differences between the early group (21.6%) and the late group (63.7%) [33]. Kurland et al. stated a rate of 5.8% for subdural effusions/hygroma and 6.8% for CSF leaks/fistulas, for an overall rate of 6.1% [36]. Malcolm et al. reported that EACs are common (62.5%) and demonstrated that early CP was the only independent predictor of EAC resolution [37]. In contrast, in our review, it only occurred in 11.8% in the early group, as opposed to only 1.10% in the late cranioplasty group.

We found one case (2%) of wound dehiscence in the early group and nine (9.90%) in the late group. In the early CP group, 55 patients (7.45%) complained of impaired wound healing, while 49 patients (6.79%) in the late group complained of wound healing. Six cases (5.45%) of wound necrosis were reported in the early group and eleven cases (8.73%) in the late group. Chasles et al. reported a combined odds ratio for wound-healing disturbance of 5% and found no differences between the groups [33]. They included the wound-healing disturbances, scalp necrosis, subcutaneous necrosis, and dehiscence in the wound-healing disturbance category. They have suggested that patients with TBI exhibit higher rates of wound healing complications, possibly because disruptions in skin continuity allow bacterial entry and predispose to wound dehiscence.

Cranioplasty reoperations necessitated by postoperative complications are associated with prolonged hospitalization, heightened surgical risk, and increased healthcare costs. We reported that reoperation was necessary in 10 patients (19.6%) receiving early CP and in six patients (6.60%) in the late CP group. In the review by Malcolm et al., the reoperation rate was relatively high at 12.9%, approaching the overall complication rate of 19.5%. Interestingly, the odds of reoperation following early CP were slightly lower than those after delayed procedures, with a trend toward statistical significance [37]. In addition, Zheng et al. found that early CP has the potential to reduce operative time and decrease dural and cortical injury because it allows easier dissection of tissue planes, as there is less scar tissue, leading to less blood loss [38].

### 4.3. Imaging and Physiological Insights

Radiological and physiological evidence support the argument for early CP. Research employing CT, PET, and transcranial Doppler has shown reversal of midline shift, ventricular dilatation, and impaired perfusion following skull restoration [12,17,23]. The findings align with experimental data indicating enhanced cerebral blood flow, glucose metabolism, and CSF dynamics following CP [7,8,23,29]. Mah et al. demonstrated with CT perfusion imaging that cerebral blood flow significantly increased both ipsilateral and contralateral to the cranial defect after CP, and these hemodynamic improvements correlated with better functional outcomes [39]. Similarly, Erdogan et al. used transcranial Doppler in eighteen patients. They found that low preoperative flow velocities on the side of the skull defect rose markedly within days after reconstruction, supporting the view that CP restores cerebral hemodynamics [40].

### 4.4. Patient Selection and Timing Considerations

Although increasing number of evidence appears in favor of early CP, timing should remain individualized. Resolution of cerebral edema, absence of infection, and clinical stability should be prerequisites [3,4,24]. Trauma patients are best served by an earlier repair [11,13,17,22], while malignant infarction or hemorrhage patients might be delayed based on systemic instability or increased risk of hydrocephalus [13,24]. Comorbidities (such as diabetes and cardiovascular disease), nutritional status, and wound healing potential should influence operative planning [16,19,21,25].

Early publications emphasize that “early” is differently defined (30–90 days) [2,16,26]. Sioutas et al. suggested stratification into ultra-early (<6 weeks), early (6 weeks–3 months), and delayed (>3 months), which could more accurately characterize outcome differences [10]. For there to be such widely used standard definitions would enhance comparability and enable firmer recommendations.

Delayed cranioplasty is widespread in real-world practice, especially in non-trauma populations. Only 44.3% of patients in the multicenter series by Fricia et al. underwent cranioplasty within 2–6 months after decompressive craniectomy, suggesting that early repair is not always possible in routine care [41].

A key aspect to keep in mind is that if the topic of CP time is limited to early versus late repair, it may be overly simple. Studies showed that the timing of cranioplasty alone does not fully determine the safety or efficacy of the procedure, as outcomes are also influenced by implant material and perioperative factors [42,43]. Ganau et al. reported that Porous Hydroxyapatite (PHA) leads to lower infection rates and better cosmetic outcomes than PolyMethyl Methacrylate (PMMA) [43]. Therefore, direct comparative studies that address the advantages and disadvantages of various cranioplasty materials may be more beneficial for advancing clinical practice than recommendations based solely on timing.

### 4.5. Limitations

The current body of evidence is limited by reliance on retrospective designs, inconsistent timing cutoffs, and heterogeneous outcome measures, which limit the strength of the evidence and introduce potential bias. [11]. Many studies have short follow-up, preventing assessment of long-term complications such as bone resorption. Single-center series predominate, limiting generalizability [13,19,21,25,26,27]. Even in recent large-scale meta-analyses, heterogeneity in patient selection, surgical technique, and outcome definitions remain as major barrier.

## 5. Conclusions

This systematic review suggests that early cranioplasty (within three months) is consistently associated with improved neurological recovery across multiple functional and cognitive domains. However, early intervention is also linked to a higher incidence of certain complications, particularly hematoma and hygroma, while delayed cranioplasty is associated with higher rates of bone flap resorption.

These findings highlight a clinically relevant trade-off: early cranioplasty may optimize neurological recovery but carries an increased risk of select complications, whereas delayed cranioplasty may offer a more stable surgical profile at the potential cost of slower functional improvement. Importantly, no included study demonstrated superior neurological outcomes with delayed intervention.

Cranioplasty timing should therefore be individualized, taking into account patient condition, underlying pathology, and perioperative factors. Beyond timing, implant material and system-related constraints may also significantly influence outcomes, underscoring the need for a multifactorial approach. Given the predominance of retrospective data and study heterogeneity, further prospective, standardized studies are required to establish definitive recommendations.

## Figures and Tables

**Table 1:** Detailed search strategy for PubMed, OVID, and WOS.

Source	Equation	Records Identified (*n*)	Filters
PubMed	*	1277	2017–2025 Language—English
OVID	*	1210	2017–2025 Language—English
Web of Science	*	1163	2017–2025 Language—English

* (Cranial defect OR cranioplasty OR decompressive craniectomy OR cranioplasty surgery). AND (traumatic brain injury OR TBI or trauma) AND (radiology OR imaging OR X-Ray OR CT OR computed tomography OR MRI OR magnetic resonance imaging OR PET OR radiographic assessment) AND (Adult* OR “18 years and older”) AND (outcome OR complication OR infection rate OR hydrocephalus OR seizure OR resorption OR hematoma OR subdural fluid OR neurological recovery OR GCS OR GOS OR GOSE OR FIM OR PGIBBD OR BI OR functional outcome) AND (early cranioplasty OR late cranioplasty OR early vs. late CP OR timing of cranioplasty).

**Table 2:** GOSE as a neurological assessment tool in patients undergoing CP after DC.

Author	Timing	Cause	Number of Patients			Gender	Mean Age	Assessment Tool	Post-Op Score	Comorbidities	Follow-Up
			Early	Late	Total						
Ozoner et al. 2020 [13]	2 Months	TBI	67	59	126	84 M, 42 F	53 51	GOSE	Early (1–4): 22 (5–8): 45 Late:(1–4): 22 (5–8): 37	Hypertension Diabetes	12 M
Vreeburg et al. 2024 [12]	3 Months	TBI	73	100	173	Early: 70 M 3 F Late: 72 M 28 F	44	GOSE	4 (2–6)	Mild and severe systemic illness	12 M

**Table 3:** GCS as a neurological assessment tool in patients undergoing CP after DC.

Authors	Etiology	Timing	Patients	Gender	Mean Age	Assessment Tool	Comorbidities	Pre-Op Score	Post-Op Score	Secondary Outcome (Midline Shift)
Berikol et al. 2023 [11]	TBI, fractures, tumors	(<1 M)	36	25 M 11 F	34.91 ± 20.62	GCS	Hypertension, Diabetes, CAD	4.61 ± 0.64	4.69 ± 0.52	0.30 ± 0.66
		(1–3 M)	32	19 M 13 F	47.12 ± 18.28		Hypertension, Diabetes, CAD, CVH	4.03 ± 0.89	4.50 ± 0.62	1.18 ± 1.46
		(3 M–6 M)	29	24 M 5 F	49.79 ± 16.10		Hypertension, Diabetes, CAD	4.13 ± 0.83	4.55 ± 0.63	1.65 ± 1.73
		(6 M–360 days)	29	16 M 13 F	42.37 ± 15.67		Hypertension, Diabetes, CVH	4.13 ± 0.74	4.58 ± 0.50	1.06 ± 2.05
		(>360 days)	12	10 M 2 F	43.33 ± 16.80		Hypertension, Diabetes, CVH	4.08 ± 0.90	4.41 ± 0.66	1.83 ± 2.75

**Table 4:** FIM as a neurological assessment tool in patients undergoing CP following DC.

Authors	Etiology	Timing	Patients	Gender	Mean Age	Assessment Tool	Pre-Op Score	Post-Op Score	Mean (SD) Difference in FIM Score	Comorbidities	Follow-Up
(Tomar et al., 2024) [20]	TBI (21, 16 M 5 F) Stroke (10, 7 M 3 F)	<3 M	16	9 M 7 F	49.50 ± 10.88	FIM	70.06 ± 18.35	77.37 ± 18.09	7.31 (5.82)	-	1 M
		>3 M	15	7 M 8 F	53.13 ± 11.5		68.40 ± 20.24	73.07 ± 23.33	4.66 (5.62)	-	1 M

**Table 5:** BI as a neurological assessment tool in patients undergoing CP following DC.

Authors	Etiology	Timing	Patients	Sex M/F	Mean Age (Range)	Assessment Tool	Comorbidities	Pre-Op Score	Post-Op Score	Follow-Up
Zhao et al., 2023 [24]	TBI	3–6 Months	58	53 M 5 F	38.81 ± 12.57 (4–65)	BI	Smoking 7, drinking 1, diabetes 6	85.77 ± 11.61	95.34 ± 9.02	12 M
		6–12 Months	42	35 M 7 F	35.38 ± 12.20 (4–65)	BI	Smoking 7 drinking 1 diabetes 6	82.74 ± 22.82	88.93 ± 22.86	12 M

**Table 6:** NIHSS, MMSE, NCSE, FIM, GCS, and stress as neurological assessment tools (6 tools) in patients undergoing CP following DC.

Author	Etiology	Timing Interval	Number of Patients	Sex M/F	Age	Assessment Tool	GCS	Pre-/Post-Op NIHSS	Post-Op FIM	MMSE	NCSE	IL-6 Cortisol TNF
Li et al., 2024 [29]	TBI	<3 Months	45	26 M 19 F	27–65	GCS, NIHS, FIM, MMS, NCSE, stress levels	13–15: 25 9–12: 18 <9: 2	22.07 ± 4.24 → 11.18 ± 2.35	35.26 ± 4.94 → 59.26 ± 6.12	18.13 ± 3.94 → 25.02 ± 4.61	52.06 ± 3.85 → 103.52 ± 10.63	4.13 IL6 diff 5.06 cortisol diff 0.91 TNF-α
		3–6 Months	45	24 M 21 F	29–64		13–15: 17 9–12: 18 <9: 9	21.49 ± 5.76 → 14.74 ± 3.61	36.15 ± 4.56 → 47.86 ± 5.27	18.69 ± 4.17 → 22.74 ± 5.13	51.97 ± 4.23 → 88.76 ± 7.39	7.79 IL6 diff 17.3 cortisol diff 2.4 TNF-α

**Table 7:** KPS, ZPS, quality of life, psychological function as neurological assessment tools (4 tools) in patients undergoing CP following DC.

Authors	Cause	Timing Interval	Number of Patients	Sex M/F	Age	Assessment Tool	Post-Op KPS	Post-Op ZPS	Psychological Function	Quality of Life
Jiang et al., 2020 [16]	TBI	4–6 weeks	28	17 M 11 F	<40: 11 >40: 17	KPS ZPS Psychological function QOL	≤50: 3 >50: 17	Poor: 3 Good: 17	Poor: 5 Good: 23	Poor: 5 Good: 23
		3–6 months	32	26 M 6 F	<40: 7 >40: 25		≤50: 17 >50: 15	Poor: 17 Good: 15	Poor: 19 Good: 13	Poor: 14 Good: 18

**Table 8:** MMSE, PGIBBD, GOS as neurological assessment tools (3 tools) in patients undergoing CP following DC.

Authors	Cause	Timing Interval	Patients	Mean Age	Assessment Tool	Pre-Op Score (Mean ± SD)	Post-Op Score (Mean ± SD)	Follow-Up
(Sharma et al., 2024) [17]	Trauma, ischemic infarct, cortical sinus venous thrombosis, intracranial hemorrhage	Early CP (<3 Months)	19	40.63 (24–58)	MMSE PGIBBD	26.11 ± 2.75 2.21 ± 0.78	28.21 ± 1.78 1.74 ± 0.61	3 Months
		Late CP (>3 Months)	19	38.53 (21–67)		23.74 ± 7.22 2.58 ± 0.90	27.00 ± 2.71 1.83 ± 0.67	3 Months
		Early CP	30	40.63	GOS	3.10 ± 0.031	4.23 ± 0.63	3 Months
		Late CP	30	38.53		3.10 ± 0.31	4.20 ± 0.81	3 Months

**Table 9:** GOS, mRS as neurological assessment tools (2 tools) in patients undergoing CP following DC.

Authors	Etiology	Timing Interval	Patients	Gender	Mean Age	Assessment Tool	GOS	mRS	Comorbidities	Follow-Up
(Aloraidi et al., 2021) [27]	TBI: 26 Infarction: 15	<3 M	41	(86 from both M) 15 F	32	GOS mRS	4 ± 1 → 4.1 ± 1	2.2 ± 1.78 → 2.2 ± 1.7	17 From both groups	315.6 Days
	TBI: 38 Infarction: 22	>3 M	60		31	GOS mRS	4 ± 1 → 4 ± 1	2.2 ± 1.78 → 2.3 ± 1.7		582.6 Days

**Table 10:** Complications as a neurological assessment tool in patients undergoing CP following DC.

Authors	Timing Cutoff Between Early and Late CP	DC Etiology	Age	Gender	Comorbidities	Total		Complications
						Early	Late	
Tomar et al. 2024 [20]	3 Months	TBI Stroke	29–66 A: 49.50 (10.88) B: 53.13 (11.50)	21 M 10 F	-	16	15	Infections, extradural hematoma, Hydrocephalus, Brone Graft Resorption
Sharma et al. 2024 [17]	3 Months	TBI ischemic infarct cortical sinus venous thrombosis intracranial hemorrhage	A: 24–58 B: 21–67 A: 40.63 B: 38.53	-	-	30	30	NONE
Aloraidi et al. 2021 [27]	3 Months	TBI Malignant Cerebral Infarction	(31.4 ± 13.9)	86 M 15 F	-	41	60	Hydrocephalus, Hygroma, Seizure Sunken Flap Syndrome, Mortality
Vyas et al. 2021 [21]	2 M	TBI	20–67 Mean: 43.7 for both	A: 38 M 6 F B: 42 M 4 F	-	44	46	Infections, Hematoma, Sunken brain, Resorption
Berikol et al. 2023 [11]	3 Months	trauma, fractures, tumors	A: 34.91 ± 20.62 B: 47.12 ± 18.28 C: 49.79 ± 16.1 D: 42.37 ± 15.67 E: 43.33 ± 16.8	A: 25 M 11 F B: 19 M 13 F C: 24 M 5 F D: 16 M 13 F E: 10 M 2 F	Hypertension, diabetes mellitus, cardiovascular disease	group 1(>1 M): 36 group 2 (1–3 M): 32	group 3, (3–6 M): 29 group 4 (6–12 M): 29 group 5 (>12 M): 12	infections, hematoma, seizure
Vreeburg et al. 2024 [12]	3 Months	TBI	24–58 Mean: 44 for both	A: 70 M 3 F B: 72 M 28 F	mild systemic illness A: 19 B: 28 severe systemic illness A: 10 B: 9	73	100	Hydrocephalus, Seizures
Safi et al. 2022 [28]	3 Months	TBI nontraum. (vasuclar/tumors/other)	41.4 ± 13.5	122 M 10 F	Diabetes mellitus, hypertension	77	55	(infections, extradural hematoma, hydrocephalus, subgaleal collections, intraparenchymal hemorrhage, wound necrosis)
Bjornson et al. 2019 [22]	3 Months	-TBI -Ischaemic stroke Intracerebral hemorrhage/SAH -Cerebral abscess	16–70 Mean: 52	63 M 27 F	-	24	66	Infection, hydrocephalus, pneumocephalus, cosmetic issues, hematoma
Tora et al. 2021 [23]	3 Months	TBI	43.7 years	A: 54 M 27 F B: 132 M 65 F	Obesity, diabetes, hypertension, smoking	51	91	infection, dehiscence, reoperation, Hydrocephalus, resorption, EAC
Zhao et al. 2023 [24]	6 Months	TBI (aSDH, EDH, combined SDH/EDH)	37 years	A: 53 M 5 F B: 35 M 7 F	Smoking drinking diabetes	58	42	EDH or SDH, wound healing complications, hydrocephalus, seizure
Ozoner et al. 2020 [13]	2 Months	TBI	53.1 ± 19.4	84 M 42 F	Hypertension, diabetes mellitus	67	59	Hydrocephalus
Lee et al. 2017 [19]	2 Months	Elevated ICP, TBI, ICH, SAH, infarct	52.6 ± 18.6	52 M 38 F	Hypertension, diabetes mellitus, liver disease, alcohol, smoking, antiplatelet use	23	50	Hemorrhage, wound infections, CSF leak, hygroma: 6 hydrocephalus
Rashidi et al. 2019 [25]	3 Months	TBI, stroke, ICH, SAH, tumors, infection	2–91 years 51.2 ± 17.0	195 M 134 F	Diabetes mellitus	70	259	infections: Group A: 3 Group B: 21
Kim et al. 2019 [15]	45 days	Aneurysmal SAH Brain tumor Cerebral infarction ICH TBI	-	80 M 46 F	-	51	87	Bone flap resorption, surgical site infection
Prasad et al. 2020 [26]	3 Months	TBI, ischemic stroke, CVT, hemorrhage	17–68 Mean: 38.3	67 M 26 F	-	78	15	Infection, Wound dehiscence, Seizures, Hematoma, Hydrocephalus, Death
Jiang et al. 2020 [16]	Early: 4–6 weeks Late: 3–6 months	TBI	17–65	A: 17 M 11 F B: 26 M 6 F		28	32	Infection, Fluid accumulation, Necrosis, Hematoma
Li et al. 2024 [29]	3 Months	TBI	24–65 A: 42.62 ± 2.64 B: 33.25 ± 3.06	A: 26 M 19 F B: 24 M 21 F	-	45	45	Infection, Hemorrhage, Necrosis, Effusion, Chewing discomfort
Chibbaro et al. 2025 [14]	1–3 Months	TBI	49 years (18–62)	2284 M 1723 F	-	Ultra early (<30 days): 352 Early (1–3 months): 1627	2028	infections, post-traumatic hydrocephalus, external hydrocephalus, seizures, epidural hematoma, subdural hematoma, subdural hygroma
Kim et al. 2025 [18]	3 Months	TBI	53.9 ± 17.4 years	-	-	31	24	Mortality not necessarily associated with the operation
Sioutas et al., 2025 [10]	3 Months	TBI	-	-	Diabetes mellitus, obesity, chronic kidney disease, asthma, heart disease	680	680	infection, ICH, SDH, hydrocephalus, meningitis/encephalitis/myelitis/encephalomyelitis, CSF leak, repeat craniectomy/craniotomy, seizures, EDH, wound disruption, abscess and granuloma, removal or replacement of bone flap/prosthetic plate, dependence on wheelchair/care provider, mortality
Yan et al., 2025 [30]	3 Months	Malignant Cerebral Infarction (MCI)	57.3 ± 6.8 years	43 M 43 F	-	37	49	ICH, infection, subcutaneous effusion, wound dehiscence, scalp necrosis, hydrocephalus, dural tear, seizure

## Data Availability

All data of this systemic analysis are available from the authors upon reasonable request.

## Supplementary Materials

The following supporting information can be downloaded at: https://www.mdpi.com/article/10.3390/jcm15082813/s1, File S1: Preferred Reporting Items for Systematic reviews and Meta-Analyses (PRISMA) Checklist [9].
